# Antibiotic prophylaxis for the prevention of fistulae in cleft palate repair: A quality improvement study

**DOI:** 10.1016/j.jpra.2024.12.003

**Published:** 2024-12-05

**Authors:** Nitisha Narayan, Suhavi Kapoor, Alistair Cobb, Neil McLean, David David, Shaheel Chummun

**Affiliations:** a Southwest Cleft Service, Bristol Dental Hospital, Lower Mauldin Street, Bristol, United Kingdom; b The Cleft Collective, Bristol Dental School, University of Bristol, Oakfield House, Oakfield Grove, Bristol, United Kingdom; c Craniofacial Australia, 205 Melbourne Street, North Adelaide, Australia

**Keywords:** Cleft palate, Antibiotic prophylaxis, Oronasal fistulae

## Abstract

**Background:**

Post-operative infection following cleft palate repair can lead to wound dehiscence and subsequent fistula formation. To prevent this, many surgeons advocate using post-operative antibiotic prophylaxis. The use of antibiotics in children is not without risks and with limited published data and variability both countrywide and in our unit, we wanted to address this research question.

**Objective:**

To assess fistula rates and whether the provision of antibiotics post-operatively affected the incidence of oronasal fistula formation in patients with cleft palate.

**Methodology:**

We performed an institutional retrospective study using data from patients undergoing primary palatoplasty between August 2021 and August 2022. These patients were divided into 2 groups. Group A included patients who received antibiotics only on induction and Group B additionally received post-operative antibiotic prophylaxis for 7 days. All participants (97) were evaluated for incidence of post-operative fistula formation.

**Results:**

There was no evidence to suggest a difference in the fistula rate between the different timings of antibiotic regimen in Cycle 1; on induction + 2 intravenous doses (Group A) P = 0.807 and 7 days post-operatively (Group B) P = 0.820. Also, in cycle 2 there was no difference in the fistula rates between the 2 groups; P = 0.546 for Group A and P = 0.571 for Group B.

**Conclusion:**

Our study suggests that the use of antibiotics post-operatively does not influence the formation of post-operative fistulae in cleft palate. This calls for a national randomised controlled study to answer this research question and achieve standardisation of practice.

## Background

Cleft lip and palate is the most common congenital anomaly in the head and neck region.^1^ The abnormal communication between the nasopharynx and the oral cavity potentially leads to altered microbiological flora, predisposing the patients to surgical site infection. Arief et al, 2005 to determine the effect of surgery (palatoplasty and lip repair) on the types and colony count of *Streptococcus* and *Staphylococcus* species in patients with cleft lip and palate,^2^ investigated the impact of palate repair on oral flora pre-and post-operatively. The main drive for this was that the overall incidence of dental caries was significantly higher for children with cleft, with a crude incidence rate ratio of 9.25 compared with the control group. Although, the development of dental caries involves multiple factors, oral microbiological flora plays a significant role in the initiation process.^3^ The study found that there was a higher pre-operative prevalence of *Staphylococcus aureus* in the oral flora of patients with cleft palate compared to those without a cleft and the colony count reduced significantly following repair of the cleft.^2^

Oronasal fistulae are a recognised complication of cleft palate repair, and these can be symptomatic or asymptomatic. The symptomatic fistulae can impact speech through air escape and/or cause nasal regurgitation of food and therefore warrant treatment. These can often be challenging to treat depending on their size, location and sometimes patient-related factors. The systematic review by Hardwicke et al in 2014 showed that the overall incidence of reported fistula globally was 8.6% (95% CI, 6.4 to 11.1%).^4^ The cause of oronasal fistula following surgery is multifactorial and includes extrinsic factors like tension, wound infection, bleeding/haematoma and ischaemic necrosis or intrinsic factors like cleft type, cleft width and systemic disease (syndromes/faltering growth).^5^ Surgical site infection can lead to wound breakdown and development of fistulae and with the evidence of a higher bacterial count in the oral flora in patients with cleft, there is potentially a higher risk for developing a wound infection and possibly a fistula. Therefore, some cleft surgeons routinely prescribe prophylactic antibiotics for primary cleft palate repair. There is, however, little published evidence to support the benefits of post-operative antibiotic use for the prevention of infection and fistula formation.^6^^,^^7^ A survey conducted among UK surgeons in the past revealed a significant lack of consensus around the perioperative use of antibiotics.^8^ The use of antibiotics in cleft surgery without evidence is not without risk, especially in the paediatric population. These include adverse drug reactions, multi-drug resistance, hypersensitivity and costs of health care services.

To promote antibiotic stewardship and the appropriate use of antibiotics in cleft palate surgery, it was decided to do a quality improvement study in our unit.

The department consists of 2 cleft surgeons (A and B). Surgeon A used intravenous co-amoxiclav on induction and then 2 I/V doses post-operatively and Surgeon B in addition to the above, prescribes oral antibiotics prophylactically for 7 days on discharge. A recently concluded study by the Cleft Collective, analysed 752 patients who underwent primary cleft palatoplasty in the UK and found that there was no evidence to suggest a difference in fistulae rates between the different timings of the antibiotic regimen (P = 0.753), (Davies et al, 2023).^9^ Based on this evidence Surgeon A changed their practice and limited the antibiotic use to only at induction. Surgeon B remained the same. We sought to assess our fistulae rates and their relationship with our 2 different antibiotics regimes and to ascertain if the change in practice had increased our fistulae rates.

## Aims

To assess fistula rate and whether the post-operative antibiotic prophylaxis regime affected the incidence of oronasal fistula formation in patients undergoing primary cleft palatoplasty.

## Methodology

This was a quality improvement study which was registered with the Trust's clinical effectiveness department. Retrospective data analysis was done using the electronic patient records and information was collected on consecutive patients undergoing primary palate repair. Relevant demographics, diagnosis, age at surgery, cleft type, antibiotic regime used, and complications were noted. Fistula formation was used as the endpoint at 3-month follow-up. In our unit, the standard post-operative follow-up is booked at 3 months from surgery, at this stage presence of an oronasal fistula in the line of the palate repair, whether it was symptomatic or not, was recorded. (D)

The unit has 2 cleft surgeons, and both perform a Sommerlad-style intravelar veloplasty (IVVP) to repair all cleft palates. They both trained in the same unit, and therefore followed the same technique. (B) However, each has a slightly different perioperative antibiotic regime. Surgeon A prefers an intravenous antibiotic on induction and a further 2 intravenous doses in the next 24 hours. Surgeon B in addition to the above, also prescribes oral antibiotics for one week after discharge, co-amoxiclav being their drug of choice. Patients were discharged on post-operative day 1. Data was collected between August 2020 and 2021 (Cycle 1) on 46 consecutive patients undergoing primary palate repair where Surgeon A prescribed antibiotics on induction and then for a further 24 hours. Surgeon B in addition prescribed antibiotics for another 7 days. In 2021, Surgeon A changed his antibiotic regime based on the study completed by the Cleft Collective, recently accepted for publication, to co-amoxiclav only on induction with no further doses of antibiotics. Surgeon B's practice remained unchanged. We then collected the same data for the following year (Cycle 2) on a further 51 patients. Chi square tests of association were used to explore associations between the exposures (antibiotic regimen) and the outcome (formation of fistula).

## Results

Data was collected for 97 consecutive patients undergoing primary palatoplasty between Aug 2020 and Aug 2022. The average age at palate repair was 16.6 months. Veau classification was used to categorise the different cleft types; Veau 1 (11.1%), Veau 2 (57.4%), Veau 3 (20.3%), Veau 4 (11.2%). This classification was used due to its ordinal nature and ease of statistical presentation. The average cleft width at the hard and soft palate junction was 10.7 mm with a standard deviation of 2.4 mm. Patients with submucous cleft palate^10^ and patients with associated syndromes were excluded.^4^ There were a total of 46 patients in Cycle 1 (27 males and 19 females) and 51 patients in Cycle 2 (31 males and 20 females).

**Figure 1:**
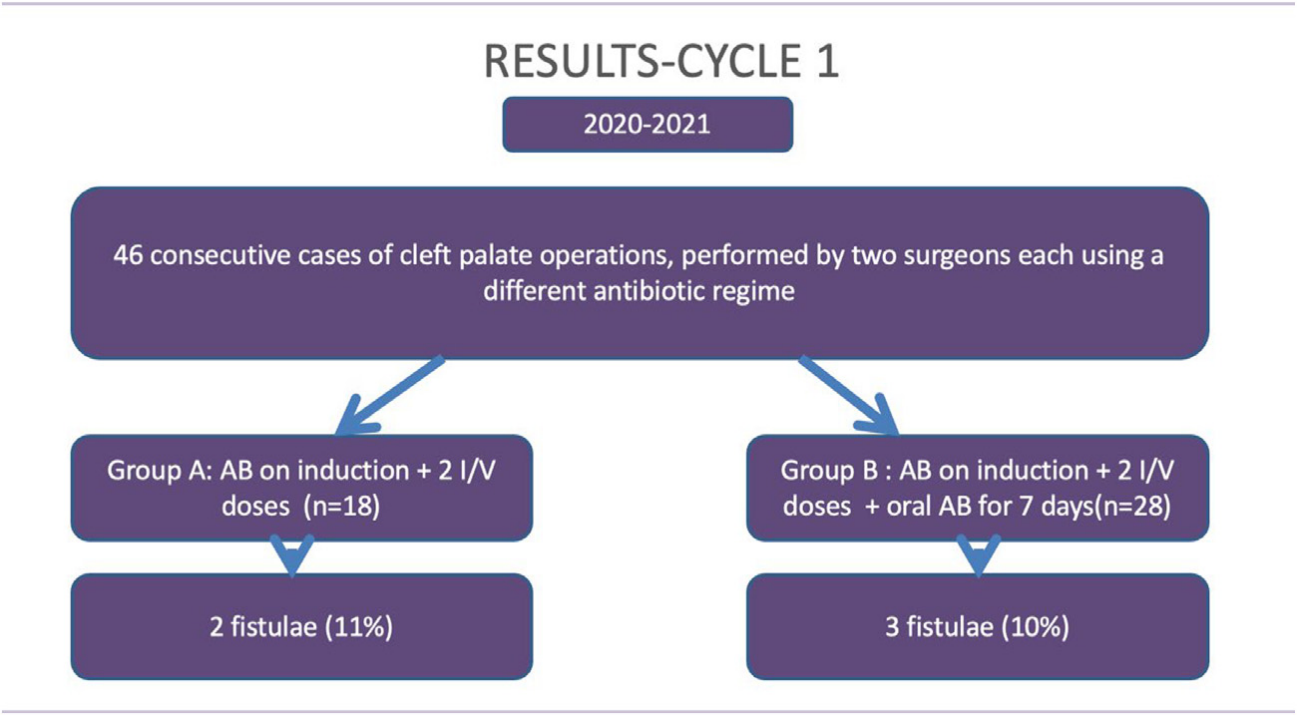
Results from Cycle 1.

**Figure 2:**
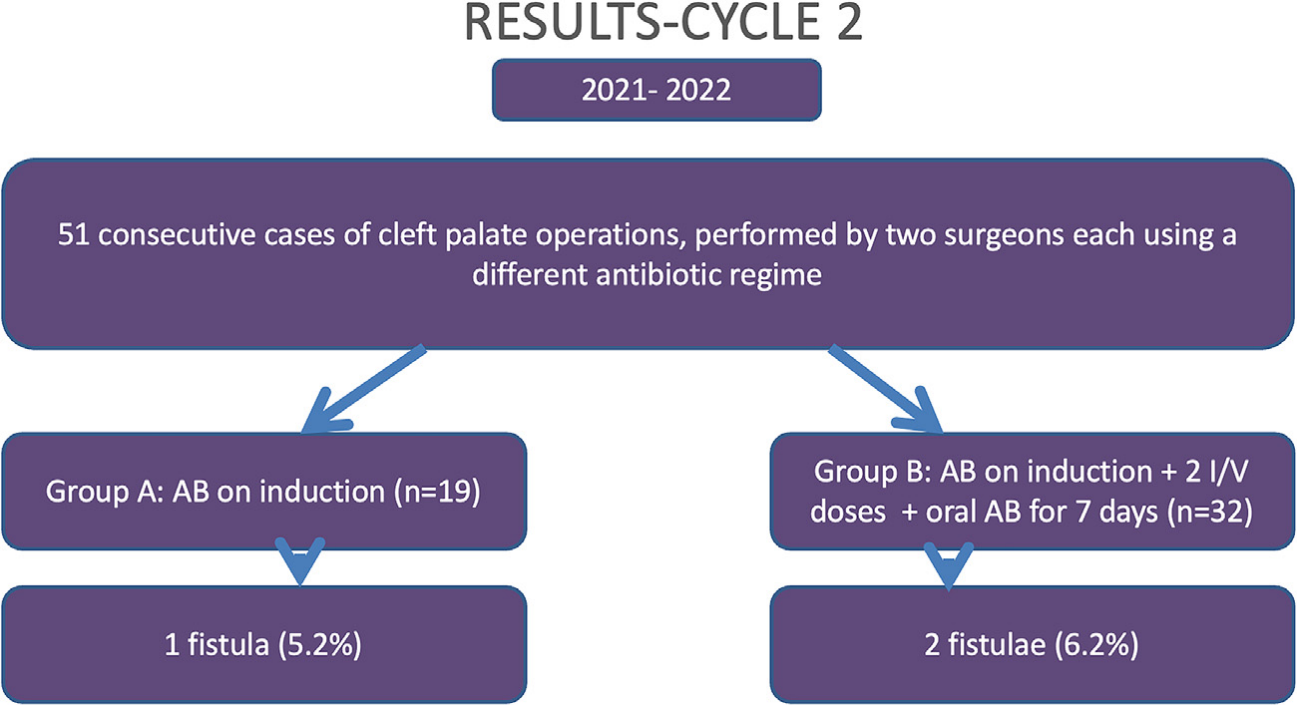
Results from Cycle 2.

All the participants received a dose of co-amoxiclav on induction. In Cycle 1 (2020-21) Group A (N = 18) received further 2 intravenous doses over the next 24 hours in hospital and Group B (N = 28) in addition, received oral antibiotics for 7 days on discharge. The presence of a fistula was documented in 5 (11.3%) patients in Cycle 1. In the first Cycle, when fistula formation was observed in the 2 groups based on the antibiotic regimes, this was 11% (2/18) for Group A and 10% (3/28) for Group B. In Cycle 2 (2021-22), Surgeon A changed his practice based on the recently concluded study from the Cleft Collective. There were 51 participants in total, 19 (Group A) received antibiotics only on induction and 32 patients (Group B) in addition received 2 intravenous doses on the ward and 7 days of oral antibiotics on discharge. The fistula rate for Cycle 2 was 5.8% (3/51) overall and was 5.2% (1/19) and 6.2% (2/32), respectively for Groups A and B. The results are summarised below for ease of understanding in Figure 1, Figure 2.

There was no evidence to suggest a difference in the fistula rate between the different timings of antibiotic regimen in Cycle 1; χ^2^  =  0.0255 P  =  0.8730, The result is not significant at p < .01. In Cycle 2 there was no difference in fistula rates between the 2 groups; χ^2^  =  0.021 P  =  0.8848.The result is not significant at p < .01. This has been summarised in Table 1.

**Table 1:** Results depicting p value between antibiotic regime used and fistula formation.

Associations between Antibiotic Regimen and the Presence of Fistula			
Cycle 1			
	Fistula Present?		Total
	No	Yes	
Antibiotic regimen			

A	16	2	18
B	25	3	28

*χ^2^ = 0.0255 P = 0.8730, The result is not significant at p < .01*			
Associations between Antibiotic Regimen and the Presence of Fistula			

Cycle 2			

	Fistula Present?		Total
	No	Yes	

Antibiotic regimen			
A	18	1	19
B	30	2	32

*χ^2^ = 0.021 P = 0.8848, The result is not significant at p < .01*			

## Discussion

This project was designed as a quality improvement study to assess our fistula rate and its relationship with the 2 antibiotic regimes used in the unit. The Cleft Collective has recently completed a UK-wide study to explore the use of peri-operative antibiotics at the time of cleft palate repair and whether this affected the rate of post-operative fistula formation.^9^ Data from the Cleft Collective was based on a sample of 752 patients from the national cohort. Out of this group, 23% were prescribed further intravenous antibiotics, and 42% were prescribed oral antibiotics for a further week. This study suggested that combining the doses of antibiotics on induction with post-operative regimens had no significant association with post-operative fistula formation. It concluded that one cannot advocate the use of antibiotics at any peri-operative stage for patients undergoing primary palatoplasty with the sole purpose of preventing post-operative fistulae.^9^

Based on this evidence, one of our surgeons changed their practice and stopped prescribing antibiotics in the post-operative period. When data was collected following this change of practice, we did not find an increase in the fistula rate in the unit.

There is a wide variability found in fistula rates reported in the literature. The fistula rate worldwide was found to be 8.6% in a meta-analysis published by Hardwicke et al in 2014.^4^ In 2016, a review published by Moar et al reported a fistula rate of 14%^10^ for the NorCleft group (Scotland and Northern England) which is comparable to Sommerlad's results and is considered by many surgeons to be the benchmark in the UK.^11^ Wound dehiscence leading to the formation of an oronasal fistula following primary palatoplasty is not an uncommon complication that is challenging for the cleft surgeon and causes emotional distress to the parents and patient.^12^ The causes of fistulae are multifactorial, including tension of wound closure and wound infection being the most common causes. Oral colonisation with pathogenic organisms could play a role in wound healing complications^13^ and it is therefore not surprising that post-operative antibiotics are prescribed following palatoplasty, to prevent wound infection and therefore dehiscence. There is however considerable variation in the choice and the post-operative antibiotic regime, used in the UK.^9^ The use of antibiotic regimes in the UK is very variable as published by Fell et al from the Cleft Collective data. They reported that out of 593 patients in the study, 32% received antibiotics only on induction, 26% received further post-op doses for 24 hrs and 42% received antibiotics for 5-7 days after discharge.^14^ In our unit there was no strong evidence for the choice of regime, but mostly what the surgeons had always trained using. (E1) A prospective randomised controlled study in India concluded that 5 days of oral antibiotics after cleft palate repair may reduce the incidence of fistulas in their study population.^15^ Another study, conducted by the John Hopkins Children's Hospital retrospectively assessed over 7 thousand children who had undergone primary palate repair and showed that the odds of having an oronasal fistula among patients who were administered pre-operative antibiotics did not differ significantly (statistically) from patients who did not receive antibiotics.^16^ Pfaff et al published a review of literature evaluating the evidence surrounding the use of peri-operative antibiotics and other antimicrobial interventions in cleft palate surgery and found that literature appears to support the use of pre-operative antibiotics for cleft palatoplasty, but the benefits of prolonged post-operative antibiotic use remain questionable.^17^ The paper also highlighted the lack of higher-level evidence on perioperative antibiotic use for cleft palatoplasty. There could be several reasons for contradictory outcomes in various studies across the globe. For example, studies conducted in developing countries often have varying age groups for palate repair, their oral microflora might be different and the choice of antibiotic used is likely different based on antibiotic sensitivities in the region. (E2)

The UK's National Institute for Health and Care Excellence (NICE) defines primary palatoplasty for cleft palate as clean-contaminated surgery and NICE recommends that antibiotic prophylaxis be given on the induction of anaesthesia. However, there is currently no guidance or standards available for post-operative prophylaxis in cleft palate surgery. Antibiotic use in children has its associated risks in the paediatric population, including adverse reactions, antibiotic resistance and additional costs on healthcare. A systematic review and meta-analysis published by Duong et al in 2022, reported that antibiotic exposure in infants was associated with an increased risk of atopic dermatitis, allergic symptoms, food allergies, allergic rhino-conjunctivitis, wheezing, asthma, increased weight gain, obesity, juvenile idiopathic arthritis, psoriasis, autism spectrum disorders and neurodevelopment disorders. These were long-term health outcomes in children, although a causal association could not be determined from these studies, the results support the meticulous application of sound antibiotic stewardship to avoid potential adverse long-term health outcomes.^18^ (F)

Our quality improvement study was designed to promote antibiotic stewardship and further supports the lack of evidence for the use of post-operative prophylactic antibiotics for cleft palate repair by demonstrating no significant difference in fistula rates between those receiving one dose of co-amoxiclav on induction and those being given the antibiotic for up to 7 days post-operatively. This study has several limitations. It was designed as a quality improvement study for 2 years in the cleft centre and therefore numbers are small. It is retrospective and the patients have not been followed up beyond 3 months. We also understand as quoted previously that multiple intra-operative factors such as cleft width, age at surgery, presence of syndromes and length of surgery may affect wound healing and occurrence of fistula. These factors have not been observed in this project and we aim to look at these factors in our future research study with larger numbers.

## Conclusion

This study demonstrates how a quality improvement study can help change practice based on scientific evidence. It suggests that the use of antibiotics post-operatively does not significantly influence the formation of post-operative fistulae in the cleft palate patient. However, a well-designed UK national randomised controlled trial is required to answer this important research question and achieve standardisation of practice.

Although this present study has small numbers, it is an important step towards promoting antibiotic stewardship in the cleft units.
